# The evolution of raw data archiving and the growth of its importance in crystallography

**DOI:** 10.1107/S205225252400455X

**Published:** 2024-06-12

**Authors:** John R. Helliwell, James R. Hester, Loes M. J. Kroon-Batenburg, Brian McMahon, Selina L. S. Storm

**Affiliations:** a Department of Chemistry University of Manchester ManchesterM13 9PL United Kingdom; b Australian Nuclear Science and Technology Organisation (ANSTO) Locked Bag 2001 Kirrawee DC New South Wales2232 Australia; c Structural Biochemistry, Bijvoet Center for Biomolecular Research Utrecht University Universiteitsweg 99 3584 CGUtrecht The Netherlands; d International Union of Crystallography 5 Abbey Square ChesterCH1 2HU United Kingdom; e European Molecular Biology Laboratory c/o DESY, Notkestraße 85 22607Hamburg Germany; Formby, Liverpool, United Kingdom

**Keywords:** raw data measuring hardware, raw data archive hardware, raw data processing software, raw data policies at photon and neutron facilities, ground truth

## Abstract

The hardware for data archiving has expanded capacities for digital storage enormously in the past decade or more. The IUCr evaluated the costs and benefits of this within an official working group which advised that raw data archiving would allow ground truth reproducibility in published studies. Consultations of the IUCr’s Commissions ensued via a newly constituted standing advisory committee, the Committee on Data. At all stages, the IUCr financed workshops to facilitate community discussions and possible methods of raw data archiving implementation. The recent launch of the *IUCrData* journal’s *Raw Data Letters* is a milestone in the implementation of raw data archiving beyond the currently published studies: it includes diffraction patterns that have not been fully interpreted, if at all. The IUCr 75th Congress in Melbourne included a workshop on raw data reuse, discussing the successes and ongoing challenges of raw data reuse. This article charts the efforts of the IUCr to facilitate discussions and plans relating to raw data archiving and reuse within the various communities of crystallography, diffraction and scattering.

## Abstract 2

The IUCr 75th Congress in Melbourne hosted a workshop on raw data reuse, continuing efforts to promote discussions and plans within crystallography, diffraction and scattering communities. These initiatives build on earlier IUCr funded workshops, aiming to establish ground truth obtained from research results through raw data archiving potential.

## 1. Introduction

A major effort of the IUCr Diffraction Data Deposition Working Group (DDDWG, 2011–2017), and now the IUCr Committee on Data (CommDat, since 2017), has been exploring the practicalities, the costs and benefits, and the opportunities for new crystallographic science arising from large-capacity data archives that have become available. We held a full-day workshop at IUCr2023 entitled ‘Raw diffraction data reuse: the good, the bad and the challenging’, bringing into focus twelve years of work in which we discussed: (1) current practices in raw data archival and sharing; (2) educating those who generate and deal in crystallographic data on best practices in data reuse in various categories of crystallographic science, with talks by leading experts; and (3) a proposed summary including the role of *IUCrData*’s new section, *Raw Data Letters*. Attendees learnt about the opportunities for raw data reuse, including the use of raw data as test datasets for machine learning, and achieving an understanding of how, and where, to effectively archive their own raw data to maximize the potential for data sharing and reuse in the future.

This workshop explored in detail the successes and challenges in practice of raw data sharing and reuse. Being a full day, it complemented the main Congress microsymposium entitled ‘Raw diffraction data reuse: warts and all’. A second microsymposium of the Committee on Data as principal proposer was entitled ‘Interoperability of Data and Databases’ (Brink *et al.*, 2024 ▸). We also secured a Congress keynote presented by Andy Götz of ESRF on the ‘European Photon and Neutron Open Science Cloud’, this being the world leading effort of this consortium of more than ten European synchrotron, neutron and X-ray laser radiation sources with raw data management and sharing.

This article has several roles. Firstly, it provides an overview of the topics addressed over the past twelve years by the IUCr’s DDDWG and Committee on Data. Secondly, it sets the scene of the international landscape on raw, processed and derived data, ensuring reproducibility of science as a whole, and thereby informs our own efforts for the best reproducibility of published crystallographic science. Thirdly, it serves as an introduction to the whole virtual issue of articles from the speakers and poster presenters of crystallographic raw data topics at IUCr2023. Fourthly, it highlights the important role of standards in the peer review of raw diffraction data, notably via enabling automated tools, which are important for ensuring standards for *Raw Data Letters* within *IUCrData*. Also, tools adopted for peer review could also ensure raw data quality at the measuring instruments such as synchrotron crystallography beamlines themselves. This latter, we hope, will also assist in increasing the fraction of published studies from measured datasets. Finally, in a ‘future vision’ section, we note that raw dataflows continue to increase substantially with improved sources and detectors along with tackling ever more challenging experiments. There is a balance to be struck therefore between compellingly good principles, such as reproducibility of published work, and the need to be pragmatic in terms of which unpublished raw data are preserved and for how long.

## 2. Truth and objectivity in crystallographic science and the role of peer review

In a scientific investigation, with one method alone, such as crystallography, we can seek as precise a result as possible, but it will inevitably, to a greater or lesser degree, be inaccurate. That degree depends on controlling as many systematic errors as possible in that one method. By using one or more complementary methods, each with a different set of systematic errors, we learn how well the several methods’ results agree, and gain insight into the accuracy overall. In the field of molecular structure science, a common example of combined methods is the analysis of crystal structures alongside NMR techniques. This theme of combining methods is explored in detail by Helliwell & Massera (2022 ▸) within two subject areas of structural molecular science: chemical and biological; the authors examine reproducibility, replicability, reliability and reusability in defining trust in a scientific study.

On submission of a publication, assessment of the validity of its results, and usually also the significance of a study, is undertaken as either pre-publication or post-publication peer review, usually both. Traditionally, the pre-publication peer review procedure involves an editor, usually known to the authors, who then consults two or more referees. Usually, the authors are not anonymous to those referees. The editor takes a final decision. These reports are usually not published. Traditionally, the post-publication peer review procedure is that a publication has a readership and individual laboratories may go so far as to first check its reproducibility and then may be inspired to design their own study to replicate and/or extend the discoveries reported in that publication. Where there are concerns these can be described in a critique article and the original authors are invited to respond. However, peer review procedures are evolving away from this traditional model. Some journals, at the point of an article’s publication, also publish the peer review reports, responses of authors and even the editor’s decision letter. Post-publication peer review can now also take the form of the article being published immediately and the readership at large having the opportunity to post comments on the journal’s website. Preprint servers have a wide role in involving the community at large as other disciplines have adopted the lead of *arXiv*, set up by physicists. The challenge for preprint servers is now to include the underpinning data, metadata and, where available, machine-generated consistency checks such as *checkCIF* or PDB validation reports in order to give the reader of a preprint a complete view of the provenance of a study.

In experimental science the effectiveness of peer review assessment is maximized by going back as far as possible in the data records underpinning those results. To borrow a term from machine learning, the raw data form the ‘ground truth’, whereas the subsequent processed data will have involved subjective choices made by the researcher in both choosing a particular software approach, and then within the software chosen. Then the derived model fit to those processed data will have involved further sets of subjective choices, again in choosing one software or another and then within the software chosen. Going back as far as possible in the recorded workflow and data files takes us as close as possible to objectivity itself. Assessment of any study benefits from a workflow record that is as complete as possible and its reproducibility assessment by a referee who is distinct from a member of the primary research team. The trend of digitally recording all steps of the experiments in electronic logbooks is an important one in this context (https://www.daphne4nfdi.de/TA1.php). There are other aspects aside from the digital ones of course, such as the choice of apparatus, beamline or detector, and the stability of their calibrations, as well as the choice of sample itself, which should also be documented.

Crystallographers are one community of several (such as astronomers and particle physicists) that have exploited digital data storage media to archive as much of their data as possible. This started with atomic coordinates; then the processed diffraction data (usually the structure factors) were added as the archiving capacity expanded. It is only in the past 15 years or so that it became practical to archive raw data. The scale of archived datasets is typically kilobytes for a file of coordinates (including their atomic displacement parameters), to megabytes for the structure factors file, to gigabytes for the zipped diffraction images file per crystal structure.

In the years since the formation of the IUCr’s Diffraction Data Deposition Working Group in 2011 and its final report delivered in 2017 (https://www.iucr.org/resources/data/dddwg/final-report), two crystal structure communities have engaged carefully, via their Commissions, in the question of the value of archiving raw diffraction images. These are the Biological Macromolecules and Structural Chemistry communities. The former firmly recommended the archiving of diffraction images for any publication communicating a new structure or a new method [for the implementation in IUCr Journals, see Helliwell *et al.*, (2019 ▸)]. By contrast, the structural chemistry community, via consultations thus far involving a questionnaire and a workshop, have reported that the procedures for raw diffraction data processing are so far satisfactory that only in extreme examples of samples with unusually challenging diffraction need their diffraction images be archived. In effect the chemical crystallographers view their ‘processed’ data, the structure factors, as their ground truth. Despite these broad consensuses, there are others in the structural chemistry community who have advocated keeping raw diffraction images nevertheless, such as for a wider application of quantum chemistry analyses (formerly known as charge density studies). In the structural biology community, there are those, not as enthusiastic about archiving diffraction images as the Commission on Biological Macromolecules, who advocate going only so far back in the data processing workflow that the unmerged structure factors are retained. The latter does allow a better diagnostic of any given experimental dataset (than merged processed data to a unique set), and its measurement timeline in particular, such as diagnosing X-ray radiation damage to the sample. It does not avoid the various subjective choices made within a raw data processing software nor the choice of which software out of several available.

Another significant community is that of powder diffractionists. The International Center for Diffraction Data (ICDD; https://www.icdd.com) have approximately half a million entries overall and include ∼10 000 raw diffraction profiles (*i.e.* without the background stripped off, rather than 2D detector diffraction images). Aranda (2018 ▸) offered views on benefits and challenges of sharing powder diffraction raw data. The Commission on Powder Diffraction is considering the possibilities in detail. Likewise, the small-angle scattering community has advanced, agreed, protocols for the management and sharing of their measured scattering data (Trewhella *et al.*, 2017 ▸).

Overall, the DDDWG recommendations can secure the best practical reproducibility of a structure derived from diffraction methods and are consistent with recommendations such as the recent USA report on best practice for *Reproducibility and Replicability in Science* (Committee on Reproducibility and Replicability in Science *et al.*, 2019 ▸).

This report, and our narrative above, does not consider unpublished data. To maximize the benefits of funds invested in science, and its facilities, scientific practice surely can and must involve maximizing the number of communicated results. This is a different issue from the reproducibility of a publication. Furthermore, measured dataflows are accelerating considerably at the new extremely bright synchrotrons and the high-data-rate electronic area detectors on beamlines (see *e.g.* Leonarski *et al.*, 2023 ▸). It is now the case that the feasibility of preserving all data in every beam time shift is under challenge. As Leonarski *et al.* (2023 ▸) neatly puts it,

X-ray facilities may have to make a difficult decision: either expand investment budget in IT infrastructure dramatically or restrict experiment performance.

Yet of course the fraction that does lead to publication is hard to predict, let alone how long to archive the unpublished raw data before taking the decision to delete. As facilities increasingly move into structural dynamics studies, quantification of the precision and accuracy of atom movements in crystal structures, both biological and chemical, will need to be ever more rigorous, not less. Since many datasets in serial crystallography contain empty frames, there is no value in storing those frames. Facility data policies such as those at the European XFEL are being revised (Dall’Antonia, 2023 ▸).

## 3. FAIR and FACT across the sciences

The wider science community is increasingly embracing the FAIR data paradigm, namely that data be Findable, Accessible, Interoperable and Reusable (Wilkinson *et al.*, 2016 ▸). Some social scientists also emphasize that more than FAIR is needed. Their data should be ‘FACT’, which is an acronym referring to Fairness, Accuracy, Confidentiality and Transparency (Van Der Aalst *et al.*, 2017 ▸). These qualities are essential to ensure reproducibility, not just reusability. This viewpoint of FAIR and FACT is similar to the view of the IUCr as to the importance of data quality and completeness (Hackert *et al.*, 2016 ▸), see below.

While FAIR looks at practical issues related to the sharing and distribution of data, FACT focuses more on the foundational scientific challenges. Although van der Aalst *et al.* (2017 ▸) write from the perspective of the social, rather than physical or biomedical sciences, there are aspects of their recommendations that apply to all sciences. In particular, the requirements for accuracy and transparency emphasize the need to work towards the highest quality in experimental data. The recent (2018) merger of the International Council for Science with the International Social Sciences Council [to form the International Science Council (ISC, 2015 ▸)] is a welcome move towards encouraging a common level of rigour across both the social and the physical/biomedical sciences.

In crystallography, the requirement for FAIR data is satisfied by our databases for processed diffraction data and their derived molecular models.

However, the FAIR data principles do not contain an explicit reference to the quality of data. The omission of this criterion by Wilkinson *et al.* (2016 ▸) may be traced to an influential OECD report of 2007, quoting from the section on quality (OECD Principles and Guidelines for Access to Research Data from Public Funding, 2007 ▸).

The value and utility of research data depends, to a large extent, on the quality of the data itself. Data managers, and data collection organizations, should pay particular attention to ensuring compliance with explicit quality standards… Although all areas of research can benefit from improved data quality, some require much more stringent standards than others. For this reason alone, universal data quality standards are not practical.

The same report also states

Where such standards do not yet exist, institutions and research associations should engage with their research community on their development.

The IUCr communities have always taken data quality seriously. As an example of a clear, overarching statement for all our crystallographic communities, the IUCr published a considered response (Hackert *et al.*, 2016 ▸) to the ISC’s report *Open Data in a Big Data World* (ISC, 2015 ▸). Within this, Hackert *et al.* (2016 ▸) noted the importance of the publication of this international accord on the values of open data in the emerging scientific culture of big data and endorsed its analysis of the value of open data. They also emphasized the generality of the accord, and went on to emphasize the crucial importance of quality control, informed by the practice within crystallography and related structural sciences:

All scientific data must be subject to rigorous first analysis to exclude or quantify systematic bias or error; all software implementations should employ open algorithmic procedures and their results should ideally be cross-checked by independent implementations. An overlooked challenge in handling ever-growing volumes of data is the need to apply the same level of critical evaluation as has been applied to historically smaller volumes…

We hold that the essential component of openness is that the data supporting any scientific assertion should be

*complete* (*i.e.* all data collected for a particular purpose should be available for subsequent re-use); and

*precise* (the meaning of each datum is fully defined, processing parameters are fully specified and quantified, statistical uncertainties evaluated and declared).

Together, these properties include the criteria… that open data should be discoverable, accessible, intelligible, assessable and usable. We note, however, that a full understanding of the data may depend on associated scientific publications that discuss the details of data processing where these differ from routine practice. The full linking of article and data is another key element of openness.

Since the publication of the FAIR principles by Wilkinson *et al.* (2016 ▸), other communities affiliated to CODATA (the ISC Committee on Data, where such matters are debated) have been pushing for a revisit of the omission of data quality in the FAIR principles. This is driven largely by the, obviously compelling, efforts of CODATA to ensure cross-domain integration of interdisciplinary data in tackling challenges such as disaster risk reduction. As an example, crystallography contributed to understanding the COVID-19 pandemic through its COVID-19 viral protein structures. These aspects of interoperability are discussed in more detail by Brink *et al.* (2024 ▸) in a companion article in this IUCr 75th Committee on Data special issue.

Let us consider how elements of the FACT criteria could be applied within the crystallographic sciences, beginning with the notion of ‘fairness’. Consider, for example, results from neutron crystallography. Because neutrons are a non-damaging probe of the structure of matter, these measurements can be made under ambient conditions, even the conditions of a living cell in terms of, say, temperature and pressure. Hence neutron crystal structures can be regarded in this sense as the closest to truth that we can reach with our atomic scale probes of the structure of matter, that also include X-rays and electrons. This statement carries the caveat that we must still work with a crystal, and it can be argued that the lattice packing arrangement may force onto a molecule some of its constituent atoms positions and/or dynamics that are not present under *in vivo* conditions. In our crystallographic databases, neutron crystal structures are by far the smallest in number due to the practical constraints of low neutron beam fluxes and long measuring times, as well as fewer instruments available globally. Not allocating proper attention to the truth of neutron crystal structures is hence ‘not fair’, to use the social scientists’ term.

Likewise, we can see the relevance of the conception of accuracy of van der Aalst *et al.* (2017 ▸) in our practices. They stress the need for

not just presenting results or make predictions, but also explicitly [providing] meta-information on the accuracy of the output.

In this context, the perception of van der Aalst *et al.* (2017 ▸) of trust is also very appropriate:

The journey from raw data to meaningful inferences involves multiple steps and actors, thus accountability and comprehensibility are essential for transparency.

The translation of this into the crystallographic sciences is perhaps best illustrated by considering structural dynamics, where changes of structure under a perturbation (such as using light in photo-crystallography) are small. Repeat processing of raw diffraction data using different software might be selected to find a structural change. Therefore, the availability of raw diffraction data allows a full comparison of results from different software, thereby establishing an estimation of the variance of atomic positions and/or *B* factors determined from those different raw diffraction data processing workflows.

The least generally applicable FACT criterion is that of confidentiality, which is most relevant to human behavioural or medical information. Nevertheless, related properties such as respecting intellectual property rights or providing access control to restricted subsets within an Open Data ecosystem (*e.g.* datasets held in a repository under a pre-publication embargo) should also be characterized by appropriate metadata within an Open Data management framework. In this ecosystem, it is summed up in the maxim that data be as open as possible and as closed as necessary (see below).

## 4. Raw data measuring hardware: sources and detectors

Let us turn now to considering the practicalities within current and planned crystallographic research areas.

In the past decade or more, major changes have been made in both source and detector capabilities. Extremely bright sources of synchrotron radiation have emerged led by MAX IV and ESRF (renamed the ESRF EBS) and similar upgrades are being applied at synchrotron facilities worldwide; the latest gains in source brightness are factors of 100 and across a wide range of photon energies. These gains are in addition to the previous frontier (low-emittance) performance of PETRA III available for the past 15 years or so. But PETRA III is now also to be upgraded to an even higher brightness PETRA IV, which will then leapfrog the ‘extremely bright sources’ yet again, promising high brilliance as well as increased flux, in particular at higher energies.

One theme for many decades in macromolecular crystallography has been to collect ‘ideal data’ using high photon energies (Helliwell *et al.*, 1993 ▸; Storm *et al.*, 2021 ▸). By exploiting the more favourable ratio of elastic scattering to photoelectric absorption at higher energies as well as detectors with high-*Z* sensor materials, datasets of higher quality can be collected (Dickerson & Garman, 2019 ▸). Nevertheless, exposure times in high-energy experiments currently need to be significantly longer than for standard energies. This is due to two main reasons. Firstly, most beamlines are not currently optimized for high energies, resulting in a significantly lower flux at higher energies. Secondly, the diffraction cross-section decreases with increasing energy, requiring a higher input of photons to obtain the same number of diffracted photons. At present, there are only very few beamlines that deliver a significant photon flux at energies higher than 20 keV and which are equipped with suitable detectors.

**Figure 1:**
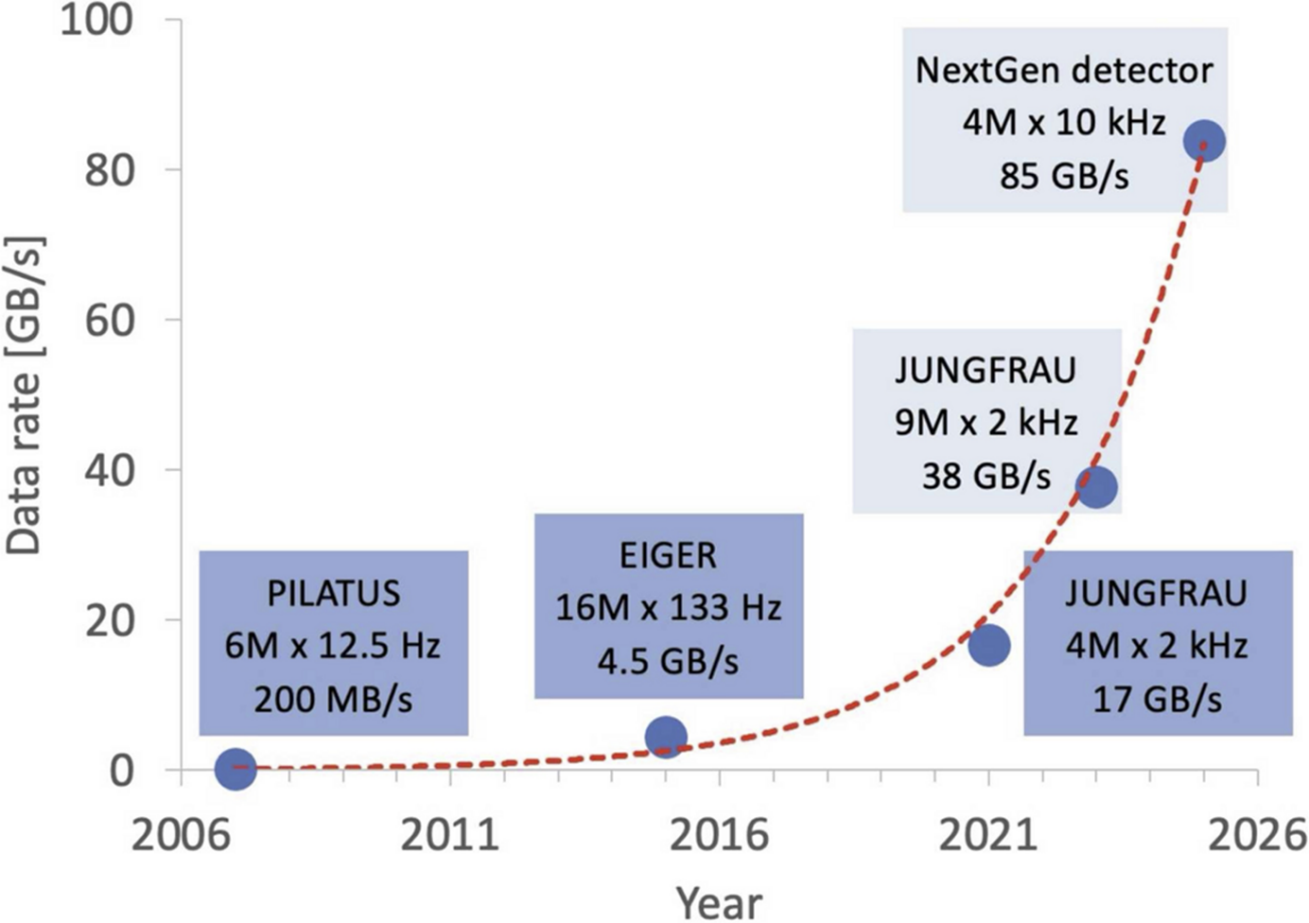
Data rates (GB s^−1^) versus calendar year of successive generations of pixel area detectors. Reproduced with the permission of Leonarski *et al.* (2023 ▸).

In another theme, smaller crystal sample volumes can be measured with a brighter X-ray beam. However, microcrystals of biological macromolecules are already viable [for a review, see Evans *et al.* (2011 ▸)] and can be investigated using a specialized beamline such as VMXm (Crawshaw *et al.*, 2022 ▸). In any case, other experimental modalities have come to the fore such as using X-ray lasers or electron crystallography, both of which yield diffraction data from sub-micrometre to nanometre sized crystals. So, a likely application of the extremely bright sources is obtaining protein structures from extremely small crystals by exploiting the photoelectron escape (Storm *et al.*, 2020 ▸) and pushing the time resolution of dynamic crystallography to shorter time intervals. This means higher data rates, provided the detectors can handle such rates; and indeed, they are currently managing to do so, as illustrated in Fig. 1 ▸.

However, can tape storage cope with such enhanced data rates? There are substantial increases in tape storage capacities planned; see Section 5. Overall, however, these enhanced data rates will challenge the current facility data archiving policies; see Section 8.

## 5. Raw data archive hardware

Since the early investigations into the topic of raw data archiving by the DDDWG (Terwilliger, 2014 ▸; Guss & McMahon, 2014 ▸), data capture rates and data volumes have increased dramatically, especially at large-scale facilities, and more attention is being given to the economic constraints on long-term archiving, as well as to the environmental impact of maintaining large server or tape storage inventories.

Prompted in part by the recommendations of the DDDWG (2017 ▸), institutional archives are reviewing their criteria for long-term retention. There is general recognition that raw diffraction datasets associated with a publication merit longer-term retention in order to satisfy the FAIR principles that support validation and reuse (Wilkinson *et al.*, 2016 ▸). The DDDWG also recommended that raw diffraction datasets for currently unsolved crystal structures, or those showing significant diffuse scattering, should also be archived. The recently launched *Raw Data Letters* section of the journal *IUCrData* (Kroon-Batenburg *et al.*, 2022 ▸) provides a vehicle for descriptive articles that should identify such datasets, and which helpfully (from the viewpoint of identifying them as candidates for long-term archiving) links them to a peer-reviewed publication.

We consider the physical repositories currently available to users in three categories: (i) general-purpose or domain repositories where users may deposit their own datasets, either because these were collected at a home laboratory or because the facility where the experiment was run does not have a satisfactory data retention policy; (ii) institutional or national repositories which collect researcher outputs voluntarily or under mandate; and (iii) the archiving systems at individual facilities.

### 5.1. User deposition in public repositories

#### 5.1.1. General-purpose repositories

Kroon-Batenburg (2019 ▸) conducted a valuable survey of raw diffraction image datasets discoverable through the OpenAire and DataCite portals. She identified a number of open access general-purpose repositories containing such datasets, as listed in Table 1 ▸.

There is clear evidence that the number of diffraction image datasets deposited in these repositories is increasing, but it is difficult to quantify the number currently held because the different repositories do not offer a suitable search filter. There does not appear to be any metadata fields that allow specification of the nature of the study with which a deposited dataset is associated.

The actual hardware stack used by these repositories is not easily found. Zenodo resides physically in the CERN Data Centre, currently using an 18 PB disk cluster. While the CERN primary storage infrastructure currently totals 150 PB of data with an expected growth of 30–50 PB per year, it is posited that Zenodo might in future move some or all of its content to offline tape storage (https://about.zenodo.org/infrastructure). Figshare runs on Amazon Web Services (https://help.figshare.com/article/figshares-approach-to-security-and-stability). Dryad data are hosted on the California Digital Library multi-campus Merritt Repository (https://datadryad.org/stash/mission). Mendeley Data also runs on Amazon Web Services (AWS) but is additionally archived with the Data Archiving and Network Services based in the Netherlands. Although additional capacity on cloud based services can usually be purchased and added easily to an existing service, it is possible that migration of content to offline tape storage might become necessary on economic grounds beyond a certain point (as already noted in the case of the locally hosted Zenodo). In the case of AWS, tape retrieval from the associated Amazon Glacier storage service may take several hours. As use of these platforms grows across different domains, their policies may change to favour disciplines or types of dataset that place the greatest demand on their services – in other words, if crystallography supplies a small proportion of their content (whether judged by storage volume, number of distinct datasets or other criteria), it may have little say in the evolution of a platform as a whole.

#### 5.1.2. Domain-specific repositories in biological crystallography

There are currently four active repositories for raw diffraction data from macromolecular crystallography experiments: the SBGrid Data Bank, the Integrated Resource for Reproducibility in Macromolecular Crystallography (IRRMC), the Xtal Raw Data Archive (XRDa) hosted by the Japanese partner of the Worldwide Protein Data Bank and the Macromolecular Xtallography Raw Data Repository (Table 2 ▸).

These archives are better suited to matching the evolving requirements of crystallographic research, and in particular their funding models might be expected to respond to the perceived needs of the active community. However, none operate on a strictly commercial basis, and so all are ultimately vulnerable to changes in public funding policy.

### 5.2. User deposition in private repositories

We use the term ‘private’ to refer to institutional or national repositories designed to store and monitor research outputs from academic staff. We do not mean to imply that the data are not made public; many institutions provide open access to deposited datasets to honour the FAIR principles such as the excellent work of the University of Manchester in this respect (Kroon-Batenburg *et al.*, 2017 ▸).

There is significant diversity in the policies and capacities of such institutions, and so we cannot draw general conclusions about their significance. Some may host copies of datasets that have also been stored in public archives. On the one hand, this increases resilience through redundancy of information; on the other hand, it complicates maintenance and raises the prospect of diverging versions.

Although these facilities increase the number of possible storage repositories, they also suffer from the shortcoming already identified, namely the difficulty in discoverability of datasets associated with specific types of research output. Many such institutions, for instance the ESRF, issue DOIs or other persistent identifiers for deposited material, so that links from the published literature do establish one ‘findable’ route. However, it is still not possible to browse or interrogate any individual repository to retrieve only datasets of a specific type (especially if they are not associated with published articles), although a cross-repository search interface is available through DataCite Commons (https://commons.datacite.org). Also archive mining tools now appear to be available (*e.g.*https://www.ch.cam.ac.uk/person/pm286) and text mining is ongoing in earnest (see *e.g.*https://core.ac.uk) as is data mining (*e.g.*http://chemdataextractor.org/docs/intro).

Many of the repositories support established protocols such as the Open Archives Initiative Protocol for Metadata Harvesting (OAI-PMH) (https://www.openarchives.org/pmh/), but we are not yet aware of any concerted efforts to introduce more granular discoverability through use of existing features within this protocol, such as the extension to the existing ‘set’ construct, as suggested by Guss & McMahon (2014 ▸).

### 5.3. Data archiving at experimental facilities

Although users of synchrotrons and other large experimental facilities will usually take copies of their collected datasets back to their home institutions, there is pressure on the facilities to offer archiving services, partly for the benefit of users who lose their own copies, or in some cases to facilitate analysis of the data with in-facility software and computing resources; but increasingly to provide repositories from which raw data may be accessed under the FAIR principles.

While facilities are generally equipped with high-performance computing platforms designed to handle the ever-growing data transfer rates of each new generation of detector, the prospect of storing large volumes of collected data for long periods is becoming increasingly challenging.

Several of the presentations at the Melbourne workshop illustrated the scale of the challenge. For example, the PETRA-III source at the German synchrotron currently stores ∼4.5 PB on disk-based (GPFS filesystem) storage over the course of a year, with a 180 day retention policy, then writes a dual tape copy (2 × 6 PB per year). The projected upgrade to PETRA-IV is anticipated to generate enough data by 2028 to require over 500 PB of disk storage and up to 1 EB (exabyte) of tape storage per year if the same data retention approach is maintained. The power requirements for data storage alone are projected to exceed 1 MW (Barty, 2023 ▸).

**Figure 2:**
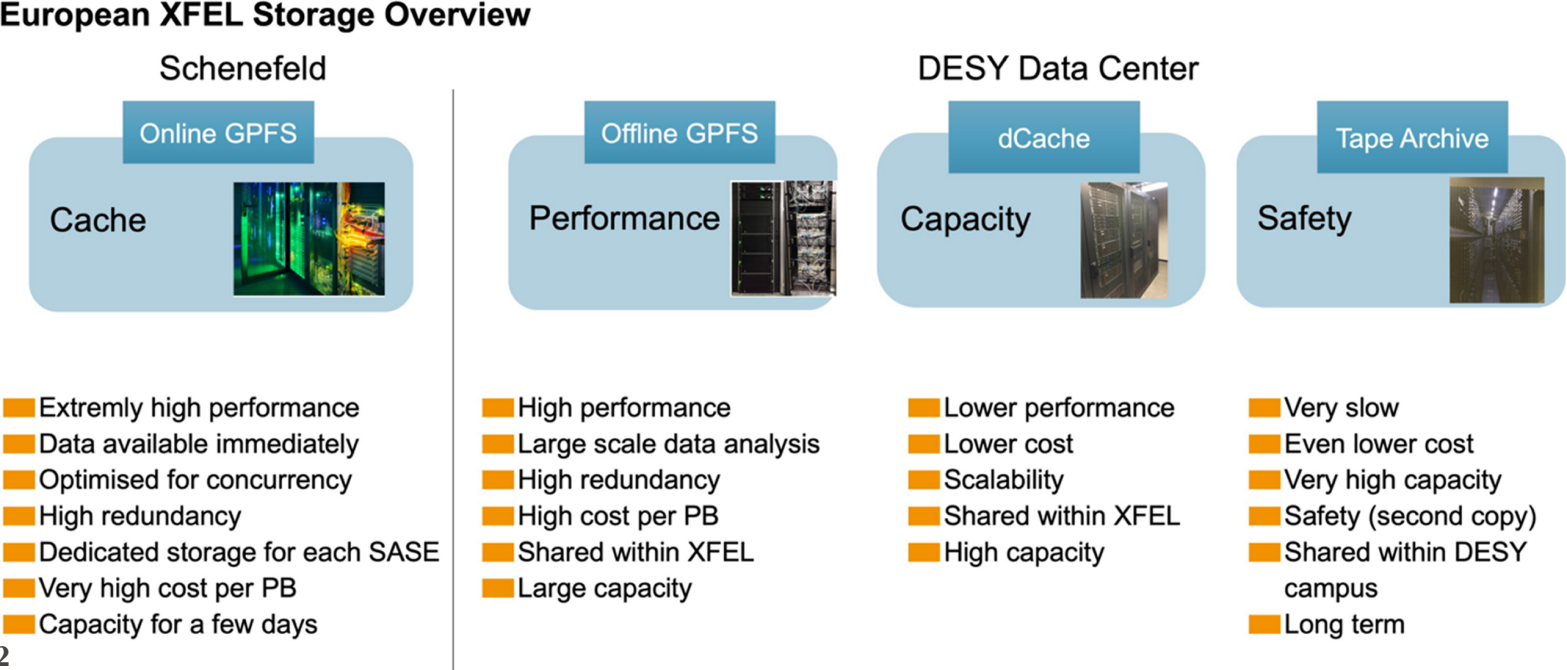
Example of a cascade of data storage approaches at the European X-FEL. Experimental data are captured in real time to very high-performance systems in Schenefeld, but progressively move via high-performance broadband to slower but higher-capacity media at the DESY Data Center as the focus shifts from processing and analysis to review, backup and potential reuse. Taken from Dall’Antonia (2023 ▸), originally created by Krzysztof Wrona, European XFEL.

Fig. 2 ▸ demonstrates the cascade of stored data to slower but higher-capacity systems at the European X-ray free-electron source at Schenefeld, which uses the DESY data centre storage infrastructure. It is notable that this model is common to many facilities, but practice is variable; for instance, at the Paul Scherrer Institute only single tape copies are retained to reduce costs (Ashton, 2023 ▸).

As pressure grows to reduce the energy footprint of large-scale facilities (Abela *et al.*, 2023 ▸), there is little doubt that more data storage will be transferred to magnetic tape. However, apart from increasing the time needed to access data stored on tape, media costs are also substantial – the PETRA-IV case study cited above projects renewables costs of EUR 50 million per year by 2028.

It therefore seems inevitable that pressure will grow on the facilities to store a smaller proportion of the raw data generated from experiments on a long-term basis. Already facilities are beginning to process raw data from serial crystallography experiments on-the-fly, and consider various strategies for retaining progressively smaller quantities of data: store hits only, store indexed frames only, use lossy compression methods, store data only when it yields results, store a random sample of the data (Tolstikova, 2023 ▸).

Note that for many facilities, crystallographic experiments do not supply the bulk of the data collected (*e.g.* in imaging experiments), so that there is a danger that the scientific desirables of the crystallographic community might come into competition with the overall economic pressures on the facility.

## 6. Raw data processing software

Most software for data processing of single-crystal data is designed for rotation scans using area detection systems. Two-dimensional detectors were developed because of the need for rapid data collection to avoid radiation damage for large unit cells in particular (Arndt & Gilmore, 1979 ▸). Processing data from the oscillation (or rotation) method presented specific problems, because of the partial nature of reflections as they move through the Ewald sphere (Rossmann *et al.*, 1979 ▸; Arndt & Gilmore, 1979 ▸). Post-refinement techniques (Rossmann, 1985 ▸) that allow the refinement of the partial nature of some reflections based on crystal orientations, beam divergence, wavelength dispersion and crystal mosaicity, their relation being detailed in papers by Greenhough & Helliwell (1982*a* ▸,*b* ▸), turned out to be very powerful. In addition, Rossmann (1985 ▸) introduced profile fitting for quantitative analyses of reflection data. Data processing includes the following steps: peak searching, indexing to find the unit cell, space group determination, determination of Bragg intensities with box summation or profile fitting and scaling to bring all reflection data on a common scale including correction for background, radiation damage and absorption correction. The workflow of crystallographic data collection and processing is different for researchers equipped with in-house diffractometers and those that use synchrotron beamlines.

Diffractometer vendors deliver fully integrated systems with sample management databases, data collection control software (often for multi-circle goniometers), data storage facilities on local computers and data processing software. The most frequently used in-house systems currently are the Bruker series of diffractometers with the *APEX/PROTEUM* data processing software based on *SAINT* (Bruker, 2019 ▸), Rigaku Oxford Diffraction diffractometers with the *CrysAlis­Pro* software (Rigaku, 2019 ▸) and Stoe diffractometers with the *X-AREA* software (Stoe & Cie, 2016 ▸). The equipment and software can be optimized for chemical and macromolecular crystallography.

For synchrotron beamlines, mainly driven by macromolecular crystallography, several software packages were developed: *Mosflm* (Leslie, 1999 ▸) mostly used via its graphical interface *iMosflm* and its incorporation in the *CCP4* program suite (Winn *et al.*, 2011 ▸); *HKL2000* (Otwinowski & Minor, 1997 ▸), which is installed at many US synchrotron beamlines; and *XDS* (Kabsch, 2010 ▸), which performs well in unattended data processing and is often used in automatic data processing pipelines. All of these packages use profile learning techniques to precisely integrate the Bragg spots. *XDS* transforms pixel data to reciprocal space to reduce experimental broadening effects in the spot profiles, making profile learning more robust and also encourages fine slicing. *EVAL* (Schreurs *et al.*, 2010 ▸) uses a ray-tracing profile simulation technique, is very versatile and capable of treating many complicated diffraction problems but has a steep learning curve. At Diamond Light Source, Lawrence Berkeley National Laboratory and CCP4, a new software suite, *DIALS*, for the analysis of crystallographic X-ray diffraction data, was developed (Winter *et al.*, 2018 ▸) which is set up in a completely modular way and built upon the *cctbx* library [*Computational Crystallography Toolbox* (Grosse-Kunstleve *et al.*, 2002 ▸)]. Users may choose any of the above packages based on their computer operating system, wish for graphical interaction with data processing or because it is installed at the synchrotron beamline.

Initially, researchers would transfer their collected data frames by slow internet connections or on DVD to their home computer and process them locally. However, in recent years the workflow has changed considerably. Most MX beamlines have developed automatic processing tools that streamline the generation of input parameters and automatically guide the user through the data reduction steps, resulting in a processed diffraction data file in .mtz format. There is no need for the user to transfer the raw images to their home computer; the .mtz file suffices. This transfers the responsibility for data archiving and curation to the synchrotron facility. Some examples of such pipelines are experiment-control and sample-management systems, such as *ISPyB* (Beteva *et al.*, 2006 ▸) now including automatic processing pipelines: *GrenADES* (Monaco *et al.*, 2013 ▸), *EDNA* (Incardona *et al.*, 2009 ▸), *AutoPROC* (Vonrhein *et al.*, 2011 ▸) at ESRF, and *xia2* (Winter, 2010 ▸) at DLS. SSRL uses *Web-Ice* (Gonzalez *et al.*, 1994 ▸) for integrated data collection and analysis.

With the advent of extremely bright sources of synchrotron radiation (see Section 4), data are collected on ever smaller crystals that easily suffer radiation damage. This is solved by multi-crystal data collection and combining the data, so-called serial synchrotron crystallography (SSX). This technique is inspired by serial femtosecond crystallography with X-ray free electron lasers (SFX), where hundreds of thousands or millions of micrometre- or even nanometre-sized crystals are irradiated and diffract before they are destroyed by radiation damage, allowing time-resolved room-temperature data collection. SFX produces a vast number of still diffraction images and new methods and software had to be developed to index the still images and correct for the intrinsic partiality of the Bragg reflections: *CrystFEL* (White *et al.*, 2012 ▸), *DIALS* (Winter *et al.*, 2018 ▸) in combination with *cctbx.xfel* (Hattne *et al.*, 2014 ▸) and *nXDS* (Kabsch, 2014 ▸). The sheer volume of data produced for a single project and per day of running the facility is enormous, and requires advanced strategies for managing and curating the data (Barty, 2023 ▸) (see Section 4).

## 7. Raw data peer review

There is no value in publishing raw data, whether in a traditional journal or through deposition in a public archive, unless the data are reusable. Reusability has two aspects: (i) correct description of the data, and (ii) sufficient information about the experimental conditions and sample from which the data have been collected. The task of the reviewer is to ensure that both of these criteria have been met.

Correct data description is largely a matter of meeting technical requirements. Journals and archives can therefore simplify the work of the raw data reviewer by limiting the range of accepted data standards and providing automated checks: much of the quality guarantee provided by the journal or archive then relies on the quality of the checking software and selection of appropriate data standards, with the reviewer providing the final assessment based on the output of automated tools.

The sample description is less susceptible to automated checks. The sample provenance needs to be sufficiently well described to repeat the experiment. Any machine-readable standard for sample description therefore needs to cover sample origins as widely varying as complex synthesis and crystallization procedures, geological field trips and bespoke industrial manufacturing equipment. Furthermore, while there are machine-readable standards covering particular sample creation procedures, it is not reasonable to automatically reject a paper if such standard information is missing, as the sample may have a novel origin not envisaged by available standards. The simplest option is therefore for the reviewer to manually assess the description of the sample for completeness, a task that is familiar from refereeing conventional papers.

Description of the experimental environment is similarly too open-ended to be covered by currently extant standards. While commonly varied parameters, such as temperature and pressure, are included in most metadata standards, specialist techniques such as pump–probe have not yet been adequately formalized. Until standards bodies catch up with developments in experimental techniques, adequate description of the sample environment will remain on the human reviewer’s checklist.

The review process for single-crystal datasets in *Raw Data Letters* addresses the above considerations in the context of a traditional journal. Dataset submissions are restricted to open standards for which required metadata have been defined, in this case files meeting either the NXmx ‘gold standard’ (Bernstein *et al.*, 2020 ▸) or equivalent imgCIF standard (Bernstein, 2006 ▸). The journal provides an online tool which generates several check images, based on which, the correctness of the geometry descriptions and wavelength can be immediately assessed. Reviewers, of course, retain the option of ingesting the dataset into their own software to determine acceptability. *Raw Data Letters* has a further requirement that the dataset have some intrinsically interesting features, which is a judgement best left to human reviewers.

In the context of public archives of raw data, curation performs a similar function to peer review for journals. The IMMRC (see Section 5.1.2 above) provides a detailed description of the data curation process (Grabowski *et al.*, 2016 ▸), which includes metadata harvesting from a variety of sources, followed by a series of checks. As IMMRC contains only structural biology datasets, which are generally linked to wwPDB depositions containing detailed sample provenance information, there is no need for the raw data deposition to include sample- or environment-related metadata.

Post-publication data review is also a viable route for raw data publications. For example, the SBGrid data archive (Meyer *et al.*, 2016 ▸) publishes datasets from registered users without assessing data reproducibility, instead reporting on data reprocessing outcomes post-publication. As for IMMRC, links to wwPDB depositions ensure that adequate descriptions of the sample have been provided.

## 8. The evolution of data policy at photon and neutron central facilities

Central facilities providing X-rays and neutrons used in crystallography, diffraction and scattering today occupy a major role. The extent of this role varies a lot between the sub-disciplines of these fields. For example, for macromolecular crystallography, around 90% of all depositions into the PDB are from synchrotron facilities. For chemical crystallography, the Cambridge Crystallographic Data Centre has approximately 98% from home laboratory X-ray source measurements. This simply reflects the much larger scattering strength of smaller unit cells seen in chemical crystallography, simpler means of crystallographic structure-factor phase determination, and thus experimental requirements that do not usually include high-intensity tuneable beams at a synchrotron beamline. However, it is important to note that the history of chemical crystallography beamlines is relatively short compared with MX. With the emergence of dedicated beamlines for chemical crystallography and materials science, the utilization of synchrotron sources in this field has significantly increased.

The evolution of photon and neutron central facilities’ data policies in the past two decades or so has seen substantial changes. These changes have reflected the practicalities as highlighted in other sections of this article. However, there is also, first of all, an evolution of policy thinking, especially by the funding agencies as they increasingly realized that some commercial publishers were making large profits out of taxpayer-funded research. Clearly this was a violation of principle not least that a member of that tax-paying public could not access the research results in scientific journals that the funding agencies as their proxies had funded. An awkward point in this simple and obviously compelling argument was that the funding agencies typically funded ‘only’ about 20% or so of the proposals put to them. So, what about the results from unfunded research? A second aspect was that many of the learned societies had their own journals that made only small surpluses which, in any case, were invested in schemes like student bursaries for their training. Nevertheless, an unstoppable momentum has built up in ensuring open access to all research results. That these results should be presented along with their underpinning data has been a tradition of crystallographers, introduced by Bragg (1913 ▸) and formalized in the crystallographic databases firstly with the Cambridge Structural Database launched in 1965 and the PDB launched in 1971. A wide spectrum of databases is available today as summarized by Bruno *et al.* (2017 ▸). A landmark in policy development was the 2007 report of the OECD, already mentioned in Section 1. Significant advances in policy development followed the publication of this report that sought to improve the practice of global science through recommendations on access to publicly funded research data. Their focus was on computer-readable data that were the primary sources for scientific research and thus appropriate for validating research findings.

In the final section on sustainability, the report states that

[sustainability] can be a difficult task, given that most research projects, and the public funding provided, have a limited duration, whereas ensuring access to the data produced is a long-term undertaking.

These guidelines from the OECD presented a challenge, as well as opportunities, to all universities, principal investigators and the central facilities. They also challenge the funding agencies who had to give a clear (or clearer) budget line to data management, storage and access costs. A common theme emerged, in mainland Europe at least, consistent with the OECD (2007 ▸) guiding principles, that

measured data will be retained by a facility for at least ten years and measured data will be made public after three years.

An overarching policy evolution is a recommended move to ‘Open Science’ led by UNESCO (https://www.unesco.org/en/open-science/about). This includes discussion of the difference between accessibility of data being FAIR, but maybe behind a journal or database paywall, *i.e.* thereby not ‘open’. The USA National Academies have made a clear distinction about unpublished raw data release being the prerogative of a principal investigator. In Europe however, the intention is release after three years of all measured data at the photon and neutron facilities. In chemical crystallography, where measurements are made at the home university in the vast majority of projects, the raw data release policy by universities is as yet unclear. In Asia the Protein Data Bank Japan have launched a companion database to PDBj for raw diffraction data, XRDa, which does not require publication. So far, XRDa provides X-ray MX, neutron MX and electron chemical crystallography raw diffraction datasets, operating on a voluntary basis.

## 9. Possible future developments: dataflows outstrip data storage capacities

With ever increasing data rates and more and more insecure funding perspectives, there might come a point in time where dataflows outstrip data storage capacities. This might be particularly true for serial crystallography experiments. As those experiments produce the most data in crystallography by far, new procedures could significantly reduce the amount of data to be stored, as outlined above. A lot can also be gained by storing the data in a compressed format. For standard crystallography, the volume of data to be stored is relatively modest compared with other disciplines such as imaging. Should one still accept the need to reduce their volume, some prerequisites should be met when realizing a more economic form for storing the results from experiments. One of them is the availability of electronic logbooks to enable full transparency on the protein’s crystallization conditions. Combining this with the realization of the gold standard for metadata (Bernstein *et al.*, 2020 ▸), it might be sufficient to only document the software applied and save the structure factors as a result of the experiment. This, of course, supposes both that accessible software archives are maintained for the long term, and that specific version snapshots can be retrieved to match the original processing workflow. However, this prevents reprocessing of data with software yet to be developed. Helliwell (2023 ▸) discussed the challenges for quantifying small structural displacements, and their error estimates, which can be the situation for structural dynamics studies.

## 10. Conclusions

Despite all the progress in instrumentation, detector technology and software, raw data still represent the ground truth. In addition, the often overlooked or under-processed raw data harbour hidden treasures, unlocking potential insights that might have been missed in the initial analysis. In MX, for example, a new step forward taken recently is making advanced exploitation of processed but unmerged reflection intensity data during processing and then model refinement (Vonrhein *et al.*, 2024 ▸). Crucially, raw data serve as a fundamental tool for training and education in the field. Providing aspiring researchers with access to the unfiltered intricacies of crystallographic experiments nurtures a deeper understanding and proficiency in the methodology.

In the realm of software development, the untapped potential within raw data emerges as a catalyst for innovation. The data may contain hidden patterns or information yet to be extracted, pushing the boundaries of what current analytical tools can reveal. To cover all these aspects appropriately, *Raw Data Letters* was founded recently.

Raw data also play a pivotal role in safeguarding against fraud. By maintaining transparency and authenticity in the data collection process, the scientific community fortifies itself against misleading or fabricated results. This becomes even more crucial in the age of artificial intelligence (AI). On the other hand, AI and machine learning in particular, offer new opportunities in the domain of raw data mining as well as text and processed data mining. In combination with electronic logbooks, the capabilities of AI could be enhanced and contribute to the reproducibility of high-quality data. This synergy propels scientific advancements and reinforces the reliability of crystallographic research.

As the activities of the DDDWG and CommDat in the past decade have demonstrated, the relevant cost–benefit analyses for archiving raw diffraction data are complex and must constantly take account of changing technologies and practices. They are also subject to available funding, which is not always under the control of the scientific community. Nevertheless, our continuing efforts to update such analyses will be important in informing public funding policies.

As the scientific landscape evolves, the discussion surrounding what to store in the context of serial crystallography becomes paramount. Continued and intensified deliberations on this front are essential for adapting to new methodologies and ensuring the seamless progression of crystallographic research.

## Figures and Tables

**Table 1:** Open-access general-purpose repositories containing raw diffraction image datasets [after Kroon-Batenburg (2019 ▸)] Additional information from https://www.re3data.org, https://www.fairsharing.org and Stall *et al.* (2023 ▸).

Repository	Description	Funding model	Fees/costs	Size limits
Zenodo	Commissioned by the European Commission (EC) and hosted by CERN. Hosts all types of research artefacts and accepts all file formats.	EC OpenAire Projects; CERN; US National Institutes of Health; Arcadia Fund; Alfred P. Sloan Foundation; donations via CERN and Society Foundation.	Free of charge	50 GB per record (higher quotas can be requested on a discretionary basis)
Figshare	Owned by Digital Science, a subsidiary of Springer Nature. Accepts data, papers, code, media and other research outputs.	Commercial; provides research infrastructure services to institutions and publishers.	Free of charge for small datasets (<20 GB)	20 GB
Figshare+	Owned by Digital Science.	Data publishing charge from depositors.	USD 240 for up to 100 GB, then USD 875 per 250 GB	20 GB–10 TB
Dryad	Non-profit membership organization. Provides a curated general-purpose repository of research data underlying scientific and medical publications.	Costs covered by institutional, publisher and funder members, otherwise a data publication charge.	One-time fee of USD 150 for authors	1 TB per dataset
Mendeley Data	Owned by Elsevier as part of Digital Commons repository family.	Subscription model for academic and government entities.	Free of charge	10 GB per dataset

**Table 2:** Open-access raw diffraction data repositories for biological crystallography

Repository	Description	Funding model	Number of datasets
SBGrid Data Bank	Community-driven repository for X-ray diffraction (also microED and lattice light-sheet microscopy) datasets in structural biology.	Subscription model for SBGrid Consortium members.	826
IRRMC	Hosted by the Minor Laboratory of University of Virginia; open to any data submissions related to structures deposited with the Protein Data Bank.	Targeted Software Development award as part of the BD2K (Big Data to Knowledge) program of the National Institutes of Health.	9852
XRDa	Aims to collect raw crystal diffraction data for entries submitted to the Protein Data Bank, as well as independent submissions. Hosted by PDBj.	Supported by National Bioscience Database Center, Japan Science and Technology Agency, and by public donations	128
MX-RDR	Archives and provides access to raw diffraction data collected for macromolecular crystals. Includes tools for creating datasets of crystallographic metadata by combining information extracted directly from diffraction images and obtained from a PDB deposit and/or user input.	Developed as a part of an EU funded project, coordinated by the Interdisciplinary Centre for Mathematical and Computational Modelling, University of Warsaw.	430

## References

[bb53] Aalst, W. M. P. van der, Bichler, M. & Heinzl, A. (2017). *Bus. Inf. Syst. Eng.* **59**, 311–313.

[bb1] Abela, R., Biscari, C., Daillant, J., Dosch, H. & Rivkin, L. (2023). *Eur. Phys. J. Plus*, **138**, 355. 10.1140/epjp/s13360-023-03947-w PMC10127963 37128294

[bb2] Aranda, M. A. G. (2018). *J. Appl. Cryst.* **51**, 1739–1744.

[bb3] Arndt, U. W. & Gilmore, D. J. (1979). *J. Appl. Cryst.* **12**, 1–9.

[bb4] Ashton, A. (2023). *Scientific computing, data sharing and reuse at PSI*, https://www.iucr.org/resources/data/commdat/melbourne-workshop#aa.

[bb5] Barty, A. (2023). *Managing and curating data flows at PETRA-IV*, https://www.iucr.org/resources/data/commdat/melbourne-workshop#aa.

[bb6] Bernstein, H. J. (2006). Vol. *G, International Tables for Crystallography*, edited by S. R. Hall & B. McMahon. pp. 199–205. Chester, England: International Union of Crystallography.

[bb7] Bernstein, H. J., Förster, A., Bhowmick, A., Brewster, A. S., Brockhauser, S., Gelisio, L., Hall, D. R., Leonarski, F., Mariani, V., Santoni, G., Vonrhein, C. & Winter, G. (2020). *IUCrJ*, **7**, 784–792. 10.1107/S2052252520008672 PMC7467160 32939270

[bb8] Beteva, A., Cipriani, F., Cusack, S., Delageniere, S., Gabadinho, J., Gordon, E. J., Guijarro, M., Hall, D. R., Larsen, S., Launer, L., Lavault, C. B., Leonard, G. A., Mairs, T., McCarthy, A., McCarthy, J., Meyer, J., Mitchell, E., Monaco, S., Nurizzo, D., Pernot, P., Pieritz, R., Ravelli, R. G. B., Rey, V., Shepard, W., Spruce, D., Stuart, D. I., Svensson, O., Theveneau, P., Thibault, X., Turkenburg, J., Walsh, M. & McSweeney, S. M. (2006). *Acta Cryst.* D**62**, 1162–1169. 10.1107/S0907444906032859 17001093

[bb9] Bragg, W. L. (1913). *Proc. R. Soc. Lond. A*, **89**, 248–277.

[bb10] Brink, A., Bruno, I., Helliwell, J. R. & McMahon, B. (2024). *IUCrJ*, **11**, 9–15. 10.1107/S2052252523010424 PMC10833386 38131388

[bb11] Bruno, I., Gražulis, S., Helliwell, J. R., Kabekkodu, S. N., McMahon, B. & Westbrook, J. (2017). *Data Sci. J.* **16**, 38.

[bb12] Committee on Reproducibility and Replicability in Science, Board on Behavioral, Cognitive and Sensory Sciences, Committee on National Statistics, Division of Behavioral and Social Sciences and Education, Nuclear and Radiation Studies Board, Division on Earth and Life Studies, Board on Mathematical Sciences and Analytics, Committee on Applied and Theoretical Statistics, Division on Engineering and Physical Sciences, Board on Research Data and Information, Committee on Science, Engineering, Medicine and Public Policy, Policy and Global Affairs, & National Academies of Sciences, Engineering and Medicine (2019). *Reproducibility and Replicability in Science*. Washington, DC: National Academies Press.

[bb13] Crawshaw, A., Warren, A., Trincao, J., Lunnon, M., Duller, G. & Evans, G. (2022). *Acta Cryst.* A**78**, e419–e419.

[bb14] Dall’Antonia, F. (2023). *Handling of big data at the European XFEL*, https://www.iucr.org/resources/data/commdat/melbourne-workshop#aa.

[bb15] DDDWG (2017). *Final report of the IUCr Diffraction Data Deposition Working Group*, https://www.iucr.org/resources/data/dddwg/final-report.

[bb16] Dickerson, J. L. & Garman, E. F. (2019). *J. Synchrotron Rad.* **26**, 922–930. 10.1107/S160057751900612X 31274414

[bb17] Evans, G., Axford, D., Waterman, D. & Owen, R. L. (2011). *Crystallogr. Rev.* **17**, 105–142.

[bb18] Gonzalez, A., Denny, R. & Nave, C. (1994). *Acta Cryst.* D**50**, 276–282. 10.1107/S0907444993013101 15299439

[bb19] Grabowski, M., Langner, K. M., Cymborowski, M., Porebski, P. J., Sroka, P., Zheng, H., Cooper, D. R., Zimmerman, M. D., Elsliger, M.-A., Burley, S. K. & Minor, W. (2016). *Acta Cryst.* D**72**, 1181–1193. 10.1107/S2059798316014716 PMC5108346 27841751

[bb20] Greenhough, T. J. & Helliwell, J. R. (1982*a*). *J. Appl. Cryst.* **15**, 493–508.

[bb21] Greenhough, T. J. & Helliwell, J. R. (1982*b*). *J. Appl. Cryst.* **15**, 338–351.

[bb22] Grosse-Kunstleve, R. W., Sauter, N. K., Moriarty, N. W. & Adams, P. D. (2002). *J. Appl. Cryst.* **35**, 126–136.

[bb23] Guss, J. M. & McMahon, B. (2014). *Acta Cryst.* D**70**, 2520–2532. 10.1107/S1399004714005185 PMC4188000 25286838

[bb24] Hackert, M. L., van Meervelt, L., Helliwell, J. R. & McMahon, B. (2016). *Open Data in a Big Data World: a Position Paper for Crystallography*, https://www.iucr.org/iucr/open-data.

[bb25] Hattne, J., Echols, N., Tran, R., Kern, J., Gildea, R. J., Brewster, A. S., Alonso-Mori, R., Glöckner, C., Hellmich, J., Laksmono, H., Sierra, R. G., Lassalle-Kaiser, B., Lampe, A., Han, G., Gul, S., DiFiore, D., Milathianaki, D., Fry, A. R., Miahnahri, A., White, W. E., Schafer, D. W., Seibert, M. M., Koglin, J. E., Sokaras, D., Weng, T.-C., Sellberg, J., Latimer, M. J., Glatzel, P., Zwart, P. H., Grosse-Kunstleve, R. W., Bogan, M. J., Messerschmidt, M., Williams, G. J., Boutet, S., Messinger, J., Zouni, A., Yano, J., Bergmann, U., Yachandra, V. K., Adams, P. D. & Sauter, N. K. (2014). *Nat. Methods*, **11**, 545–548. 10.1038/nmeth.2887 PMC4008696 24633409

[bb26] Helliwell, J. R. (2023). *Curr. Res. Struct. Biol.* **6**, 100111. 10.1016/j.crstbi.2023.100111 PMC10695842 38058355

[bb27] Helliwell, J. R., Ealick, S., Doing, P., Irving, T. & Szebenyi, M. (1993). *Acta Cryst.* D**49**, 120–128. 10.1107/S0907444992006747 15299553

[bb28] Helliwell, J. R. & Massera, C. (2022). *J. Appl. Cryst.* **55**, 1351–1358. 10.1107/S1600576722007208 PMC9533758 36249510

[bb29] Helliwell, J. R., Minor, W., Weiss, M. S., Garman, E. F., Read, R. J., Newman, J., van Raaij, M. J., Hajdu, J. & Baker, E. N. (2019). *J. Appl. Cryst.* **52**, 495–497. 10.1107/S1600576719005922 PMC6557178 31236090

[bb30] Incardona, M.-F., Bourenkov, G. P., Levik, K., Pieritz, R. A., Popov, A. N. & Svensson, O. (2009). *J. Synchrotron Rad.* **16**, 872–879. 10.1107/S0909049509036681 19844027

[bb31] International Science Council (2015). *Open Data in a Big Data World*, https://council.science/publications/open-data-in-a-big-data-world.

[bb32] Kabsch, W. (2010). *Acta Cryst.* D**66**, 125–132. 10.1107/S0907444909047337 PMC2815665 20124692

[bb33] Kabsch, W. (2014). *Acta Cryst.* D**70**, 2204–2216. 10.1107/S1399004714013534 PMC4118830 25084339

[bb34] Kroon-Batenburg, L. (2019). *Raw Data Opportunities for Biological Crystallography Publishing*, https://www.iucr.org/__data/assets/pdf_file/0018/144009/07_KroonBatenburg_Rawdata.pdf.

[bb35] Kroon-Batenburg, L. M. J., Helliwell, J. R. & Hester, J. R. (2022). *IUCrData*, **7**, x220821. 10.1107/S2414314622008215 PMC9635430 36337453

[bb36] Kroon-Batenburg, L. M. J., Helliwell, J. R., McMahon, B. & Terwilliger, T. C. (2017). *IUCrJ*, **4**, 87–99. 10.1107/S2052252516018315 PMC5331468 28250944

[bb37] Leonarski, F., Nan, J., Matej, Z., Bertrand, Q., Furrer, A., Gorgisyan, I., Bjelčić, M., Kepa, M., Glover, H., Hinger, V., Eriksson, T., Cehovin, A., Eguiraun, M., Gasparotto, P., Mozzanica, A., Weinert, T., Gonzalez, A., Standfuss, J., Wang, M., Ursby, T. & Dworkowski, F. (2023). *IUCrJ*, **10**, 729–737. 10.1107/S2052252523008618 PMC10619449 37830774

[bb38] Leslie, A. G. W. (1999). *Acta Cryst.* D**55**, 1696–1702. 10.1107/s090744499900846x 10531519

[bb39] Meyer, P. A., Socias, S., Key, J., Ransey, E., Tjon, E. C., Buschiazzo, A., Lei, M., Botka, C., Withrow, J., Neau, D., Rajashankar, K., Anderson, K. S., Baxter, R. H., Blacklow, S. C., Boggon, T. J., Bonvin, A. M. J. J., Borek, D., Brett, T. J., Caflisch, A., Chang, C.-I., Chazin, W. J., Corbett, K. D., Cosgrove, M. S., Crosson, S., Dhe-Paganon, S., Di Cera, E., Drennan, C. L., Eck, M. J., Eichman, B. F., Fan, Q. R., Ferré-D’Amaré, A. R., Christopher Fromme, J., Garcia, K. C., Gaudet, R., Gong, P., Harrison, S. C., Heldwein, E. E., Jia, Z., Keenan, R. J., Kruse, A. C., Kvansakul, M., McLellan, J. S., Modis, Y., Nam, Y., Otwinowski, Z., Pai, E. F., Pereira, P. J. B., Petosa, C., Raman, C. S., Rapoport, T. A., Roll-Mecak, A., Rosen, M. K., Rudenko, G., Schlessinger, J., Schwartz, T. U., Shamoo, Y., Sondermann, H., Tao, Y. J., Tolia, N. H., Tsodikov, O. V., Westover, K. D., Wu, H., Foster, I., Fraser, J. S., Maia, F. R. N. C., Gonen, T., Kirchhausen, T., Diederichs, K., Crosas, M. & Sliz, P. (2016). *Nat. Commun.* **7**, 10882. 10.1038/ncomms10882 PMC4786681 26947396

[bb40] Monaco, S., Gordon, E., Bowler, M. W., Delagenière, S., Guijarro, M., Spruce, D., Svensson, O., McSweeney, S. M., McCarthy, A. A., Leonard, G. & Nanao, M. H. (2013). *J. Appl. Cryst.* **46**, 804–810. 10.1107/S0021889813006195 PMC3654316 23682196

[bb41] OECD Principles and Guidelines for Access to Research Data from Public Funding (2007). https://www.oecd.org/science/inno/38500813.pdf.

[bb42] Otwinowski, Z. & Minor, W. (1997). *Methods Enzymol.* **276**, 307–326. 10.1016/S0076-6879(97)76066-X 27754618

[bb43] Rigaku (2019). *CrysAlisPro*. Rigaku Oxford Diffraction Ltd, Yarnton, Oxfordshire, England.

[bb44] Rossmann, M. G., Leslie, A. G. W., Abdel-Meguid, S. S. & Tsukihara, T. (1979). *J. Appl. Cryst.* **12**, 570–581.

[bb101] Rossmann, M. (1985). *Methods Enzymol.* **14**, 237–280. 10.1016/0076-6879(85)14022-x 4079769

[bb45] Schreurs, A. M. M., Xian, X. & Kroon-Batenburg, L. M. J. (2010). *J. Appl. Cryst.* **43**, 70–82.

[bb46] Stall, S., Martone, M. E., Chandramouliswaran, I., Federer, L., Gautier, J., Gibson, J., Hahnel, M., Larkin, J., Pfeiffer, N., Sedora, B., Sim, I., Smith, T., Van Gulick, A. E., Walker, E., Wood, J., Zaringhalam, M. & Zigoni, A. (2023). Generalist Repository Comparison Chart (3.0). https://doi.org/10.5281/zenodo.7946938.

[bb47] Stoe & Cie (2016). *X-AREA* software suite. Stoe & Cie, Darmstadt, Germany.

[bb48] Storm, S. L. S., Axford, D. & Owen, R. L. (2021). *IUCrJ*, **8**, 896–904. 10.1107/S2052252521008423 PMC8562668 34804543

[bb49] Storm, S. L. S., Crawshaw, A. D., Devenish, N. E., Bolton, R., Hall, D. R., Tews, I. & Evans, G. (2020). *IUCrJ*, **7**, 129–135. 10.1107/S2052252519016178 PMC6949606 31949913

[bb50] Terwilliger, T. C. (2014). *Acta Cryst.* D**70**, 2500–2501. 10.1107/S139900471402118X PMC8494196 25286835

[bb51] Tolstikova, A. (2023). *Processing data in serial crystallography on-the-fly: what kind of raw data do we want to store?*https://www.iucr.org/resources/data/commdat/melbourne-workshop#at

[bb52] Trewhella, J., Duff, A. P., Durand, D., Gabel, F., Guss, J. M., Hendrickson, W. A., Hura, G. L., Jacques, D. A., Kirby, N. M., Kwan, A. H., Pérez, J., Pollack, L., Ryan, T. M., Sali, A., Schneidman-Duhovny, D., Schwede, T., Svergun, D. I., Sugiyama, M., Tainer, J. A., Vachette, P., Westbrook, J. & Whitten, A. E. (2017). *Acta Cryst.* D**73**, 710–728. 10.1107/S2059798317011597 PMC5586245 28876235

[bb54] Vonrhein, C., Flensburg, C., Keller, P., Fogh, R., Sharff, A., Tickle, I. J. & Bricogne, G. (2024). *Acta Cryst.* D**80**, 148–158. 10.1107/S2059798324001487 PMC10910543 38411552

[bb55] Vonrhein, C., Flensburg, C., Keller, P., Sharff, A., Smart, O., Paciorek, W., Womack, T. & Bricogne, G. (2011). *Acta Cryst.* D**67**, 293–302. 10.1107/S0907444911007773 PMC3069744 21460447

[bb56] White, T. A., Kirian, R. A., Martin, A. V., Aquila, A., Nass, K., Barty, A. & Chapman, H. N. (2012). *J. Appl. Cryst.* **45**, 335–341.

[bb57] Wilkinson, M. D., Dumontier, M., Aalbersberg, Ij. J., Appleton, G., Axton, M., Baak, A., Blomberg, N., Boiten, J.-W., Da Silva Santos, L. B., Bourne, P. E., Bouwman, J., Brookes, A. J., Clark, T., Crosas, M., Dillo, I., Dumon, O., Edmunds, S., Evelo, C. T., Finkers, R., Gonzalez-Beltran, A., Gray, A. J. G., Groth, P., Goble, C., Grethe, J. S., Heringa, J., ’T Hoen, P. A. C., Hooft, R., Kuhn, T., Kok, R., Kok, J., Lusher, S. J., Martone, M. E., Mons, A., Packer, A. L., Persson, B., Rocca-Serra, P., Roos, M., Van Schaik, R., Sansone, S.-A., Schultes, E., Sengstag, T., Slater, T., Strawn, G., Swertz, M. A., Thompson, M., Van Der Lei, J., Van Mulligen, E., Velterop, J., Waagmeester, A., Wittenburg, P., Wolstencroft, K., Zhao, J. & Mons, B. (2016). *Sci. Data*, **3**, 160018.

[bb58] Winn, M. D., Ballard, C. C., Cowtan, K. D., Dodson, E. J., Emsley, P., Evans, P. R., Keegan, R. M., Krissinel, E. B., Leslie, A. G. W., McCoy, A., McNicholas, S. J., Murshudov, G. N., Pannu, N. S., Potterton, E. A., Powell, H. R., Read, R. J., Vagin, A. & Wilson, K. S. (2011). *Acta Cryst.* D**67**, 235–242. 10.1107/S0907444910045749 PMC3069738 21460441

[bb59] Winter, G. (2010). *J. Appl. Cryst.* **43**, 186–190.

[bb60] Winter, G., Waterman, D. G., Parkhurst, J. M., Brewster, A. S., Gildea, R. J., Gerstel, M., Fuentes-Montero, L., Vollmar, M., Michels-Clark, T., Young, I. D., Sauter, N. K. & Evans, G. (2018). *Acta Cryst.* D**74**, 85–97. 10.1107/S2059798317017235 PMC5947772 29533234

